# A Vesicular Stomatitis Virus‐Inspired DNA Nanocomplex for Ovarian Cancer Therapy

**DOI:** 10.1002/advs.201700263

**Published:** 2017-12-29

**Authors:** Wei Zhao, Yuping Yang, Lingling Song, Tianyi Kang, Ting Du, Yujiao Wu, Meimei Xiong, Li Luo, Jianlin Long, Ke Men, Lan Zhang, Xiaoxin Chen, Meijuan Huang, Maling Gou

**Affiliations:** ^1^ State Key Laboratory of Biotherapy and Cancer Center West China Hospital Sichuan University, and Collaborative Innovation Center for Biotherapy Chengdu 610041 P. R. China; ^2^ Department of Thoracic Oncology Cancer Center and State Key Laboratory of Biotherapy West China Hospital West China Medical School Sichuan University Chengdu 610041 P. R. China; ^3^ Community Health Service Administration Center Shenzhen Longhua New District Central Hospital Shenzhen 518110 P. R. China; ^4^ Research and Development Department Guangdong Zhongsheng Pharmacy Dongguan 523325 China

**Keywords:** bioinspiration, cancer therapy, gene delivery, nanoparticles, ovarian cancer

## Abstract

Gene therapy provides a novel method for cancer therapy. This study shows a DNA nanocomplex that is inspired from vesicular stomatitis virus (VSV) for ovarian cancer therapy. This DNA nanocomplex consists of a cationized monomethoxy poly (ethylene glycol)‐poly (d,l‐lactide) (MPEG‐PLA) nanoparticle and a plasmid encoding the matrix protein of vesicular stomatitis virus (VSVMP) that plays a critical role in the VSV‐induced apoptosis of cancer cells. The cationized MPEG‐PLA nanoparticle that is self‐assembled by MPEG‐PLA copolymer and ***N***‐[1‐(2,3‐dioleoyloxy) propyl]‐*N,N,N*‐trimethylammonium chloride (DOTAP) has low cytotoxicity and high transfection efficiency (>80%). Intraperitoneal administration of the p***VSVMP*** nanocomplex remarkably inhibits the intraperitoneal metastasis of ovarian cancer and does not cause significant systemic toxicity. The apoptosis induction and anti‐angiogenesis are involved in the anticancer mechanism. This work demonstrates a VSV‐inspired DNA nanocomplex that has potential application for the treatment of intraperitoneal metastasis of ovarian cancer.

## 1 Introduction

Epithelial ovarian carcinoma is the major cause of gynecologic cancer‐related death.^1^ Currently, the combination of surgical cytoreduction with subsequent platinum/taxane cytotoxic chemotherapy is the preferred therapeutic regimen for ovarian cancer therapy.^2^ However, the overall five‐year survival rate of advanced ovarian cancer (stage IIIC or IV [FIGO]) is less than 25% due to the drug resistance and cancer recurrence.[[qv: 2b,3]] Therefore, novel therapeutic strategies for ovarian cancer are under urgent desire.

Vesicular stomatitis virus (VSV), a negative‐stranded RNA rhabdovirus with a single genome encoding five proteins (N, P, M, G, and L), can preferentially replicate in malignant cells and eventually induce cell apoptosis.^4^ However, the VSV still has several limitations for cancer therapy in clinical application, which can cause a series of severe side effects, including flu‐like symptoms, oral vesicles, neuropathogenesis, or cervical lymphadenopathy.[[qv: 4c,5]] These virus‐associated safety issues severely restrict the clinical application of VSV for cancer treatment.

Gene therapy provides a strategy for cancer treatment via regulating a variety of cellular activities including DNA repair, cell cycle arrest, mitogenic signaling, cell differentiation, migration, and programmed cell death.^6^ The matrix protein of VSV (VSVMP), a structural component of the virion, plays a critical role in inducing visible cytopathic effects in the absence of other viral structural components.[[qv: 4a,7]] The cytopathic effects induced by VSVMP can cause the destruction of all the three types of cytoskeletal elements (actin, vimentin, and tubulin)^8^ and the inhibition of gene expression in the host cell,^9^ inspiring that VSVMP could be applied in cancer gene therapy. However, the lack of efficient and safe delivery systems becomes one of the major obstacles in gene therapy.^10^

**Scheme 1:**
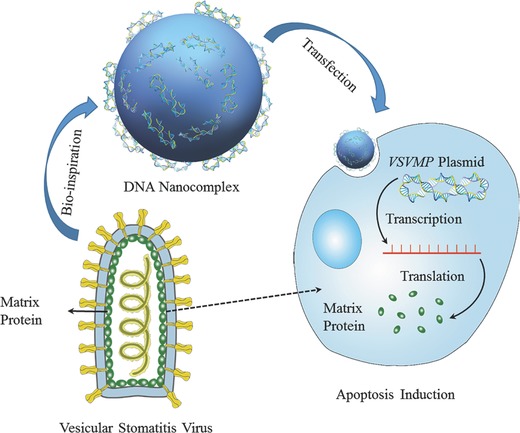
VSV‐inspired DNA nanocomplex for ovarian cancer therapy. The designed VSV‐inspired DNA nanocomplex consists of a cationized MPEG‐PLA nanoparticle complexed with p*VSVMP*. The p*VSVMP* nanocomplex can efficiently express the matrix protein into SKOV3 ovarian cancer cells and eventually lead to apoptosis‐induced cell death. Furthermore, intraperitoneal administration of p*VSVMP* nanocomplex can efficiently inhibit intraperitoneal metastatic ovarian cancer without VSV‐associated safety issues.

Recently nonviral vectors in gene therapy have attracted an increasing attention because of several advantages, such as exempt of endogenous virus recombination, nonimmunogenicity, simplicity in usage, ease of large‐scale production, and efficient delivery capacity of genetic materials.[[qv: 10b,11]] In this study, we design a VSV‐inspired DNA nanocomplex for ovarian cancer therapy (**Scheme** **1**). The DNA nanocomplex is composed of *VSVMP* plasmid and biodegradable cationic MPEG‐PLA nanoparticle. In vitro results indicate that this nanocomplex can efficiently deliver VSVMP gene into ovarian cancer cells. Intraperitoneal administration of p*VSVMP* nanocomplex can significantly inhibit the intraperitoneal metastasis of ovarian cancer via induction of apoptosis and anti‐angiogenesis, and does not cause obvious systemic toxicity. Our data suggest that the VSV‐inspired p*VSVMP* nanocomplex has great potential for clinical application in the therapy of intraperitoneal metastasis of ovarian cancer.

## 2 Results

### 2.1 Preparation and Characterization of the DNA Nanocomplex

**Figure 1:**
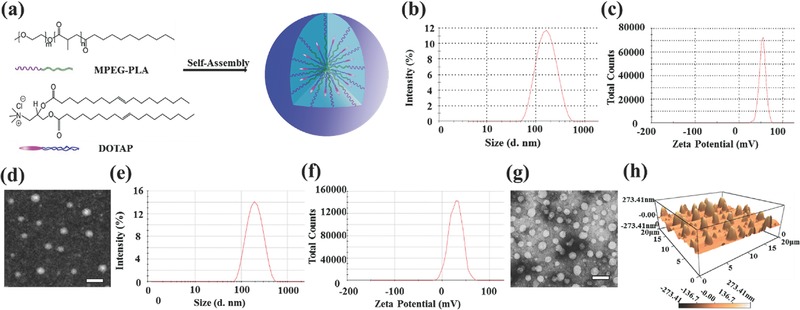
Characterization of CNPs and DNA nanocomplex. a) Schematic of the prepared procedure of the CNPs. b) Size distribution spectrum and c) zeta potential spectrum of the CNPs determined by Zetasizer Nano ZS. d) Morphologic feature of CNPs detected by TEM. Scale bar, 200 nm. e) Size distribution spectrum and f) zeta potential spectrum of the DNA nanocomplex determined by Zetasizer Nano ZS; the morphologic feature of DNA nanocomplex detected by g) TEM (scale bar, 200 nm) and h) AFM.

**Figure 2:**
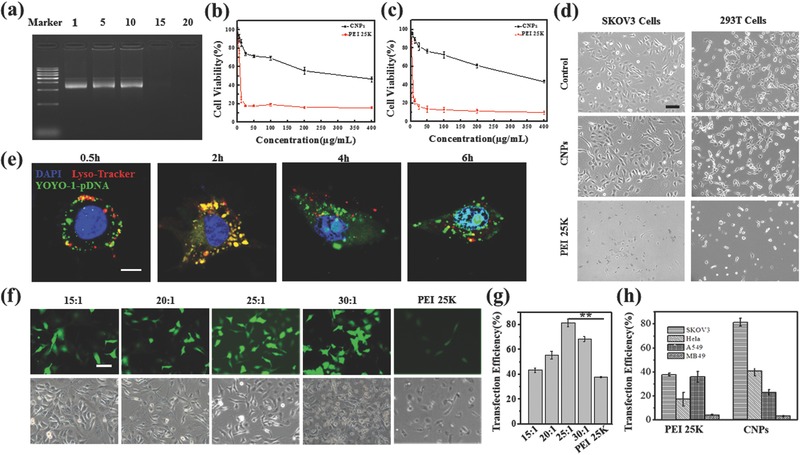
The activity of DNA nanocomplex in vitro. a) The DNA‐binding capacity of the CNPs determined by gel retardation assay. Panels (b) and (c) are the cytotoxicity of CNPs to SKOV3 cells and 293T cells determined by MTT assay, respectively. d) Cytotoxicity of CNPs to SKOV3 cells and 293T cells observed under microscopy. Scale bar, 100 µm. e) Confocal images of SKOV3 cells treated with p*VSVMP* nanocomplex for 0.5, 2, 4, and 6 h. p*VSVMP* was labeled with YOYO‐1(green), the endosomes and lysosomes were stained with LysoTracker Red (red), and the nuclei were stained with DAPI (blue). Scale bar, 20 µm. f) GFP‐derived green fluorescence image (up panels) and Brilliant image (down panels) of SKOV3 cells were observed under fluorescent microscopy after being treated with PEI25K/pGFP or different concentration of CNPs/pGFP. Scale bar, 100 µm. g) Quantitative analysis of GFP‐positive cells (%) of different concentration of CNPs/pGFP and PEI25K/pGFP. h) Quantitative analysis of GFP‐positive cells (%) of CNPs/pGFP and PEI25K/pGFP in different cell lines. **P < 0.01.

To develop a nonviral gene delivery system for cancer gene therapy, we used *N*‐[1‐(2,3‐dioleoyloxy) propyl]‐*N,N,N*‐trimethylammonium chloride (DOTAP) and monomethoxy poly (ethylene glycol)‐poly (d,l‐lactide) (MPEG‐PLA) to self‐assemble into stable cationized nanoparticles (CNPs), as shown in **Figure** **1**a. The distribution spectrum of particle size (Figure 1b) indicated that the CNPs had a hydrodynamic size of 182 ± 6 nm. The zeta potential of the CNPs was +46 ± 2 mV (Figure 1c). Morphologic feature determined by transmission electron microscopy (TEM) image (Figure 1d) showed that these particles had a mean size of 73 ± 7 nm. The particle size measured by dynamic light scattering and transmission electron microscopy indicated that the CNPs have a good water distribution.^12^ We further investigated the distribution spectrum of particle size and zeta potential of DNA nanocomplex. It showed a moderate increase in particle size as 194 ± 16 nm (Figure 1e) without significant difference compared with the CNPs (*P* = 0.39) while a decrease as 28 ± 2 mV in the zeta potential (Figure 1f). The transmission electron microscopy (TEM) showed that the DNA nanocomplex had a sphere morphology with a mean particle size of 82 ± 7 nm (Figure 1g). As shown in Figure 1h, the atomic force microscope (AFM) image indicated that the DNA nanocomplex also had a similar particle size as null CNPs. A gel retardation assay was performed to evaluate the DNA‐binding ability of CNPs. When the mass ratio of CNPs to DNA was 15: 1, the anionic DNA was totally retarded (**Figure** **2**a). A 3‐(4,5‐dimethylthiazol‐2‐yl)‐2,5‐diphenyl tetrazolium bromide (MTT) assay was performed to investigate cytotoxicity of the transfection materials (CNPs and PEI25K). It showed that the IC_50_ value of CNPs in SKOV3 (Figure 2b, IC_50_ = 316.0 µg mL^−1^) and 293T (Figure 2c, IC_50_ = 338.2 µg mL^−1^) were significantly higher than that of PEI25K (10 µg mL^−1^), implying that CNPs had low cytotoxicity (Figure 2d).

We explored the intracellular trafficking of DNA nanocomplex labeled by YOYO‐1 in SKOV3 cells in a time‐dependent manner. We observed the colocalization of YOYO‐1‐pDNA nanocomplex (green) and endosomal/lysosomal (red) after incubating for 2 h (Figure 2e). After 4 h of incubation, the DNA nanocomplex escaped from the endosomes effectively and entered into the nuclei. The result indicated that the intracellular transfection of DNA nanocomplex might be through endosomal/lysosomal pathway.

To detect the gene expression of the nanocomplex, the mass ratio of CNPs:DNA from 15:1 to 30:1 was used to formulate the DNA nanocomplex. GFP plasmid was used as a report gene (pGFP). As shown in Figure 2f,g, the CNP/pGFP nanocomplex at the mass ratio of 25:1 showed the highest transfection efficiency (81.3% ± 3.2%), which was also higher than that of the PEI25K as a gold standard transfection reagent (37.7% ± 0.6%). We also investigated the transfection efficiency (CNPs vs PEI25K) on HeLa cells (40.5% ± 3.9% vs 17.3% ± 5.7%), A549 cells (22.8% ± 2.4% vs 35.9% ± 4.5%), and MB49 cells (3.2% ± 0.5% vs 4% ± 0.6%) (Figure 2h). These results indicated that the CNP nanocomplex had an excellent transfection efficiency in SKOV3 ovarian cancer cells.

### 2.2 In Vitro Anticancer Activity of the DNA Nanocomplex

**Figure 3:**
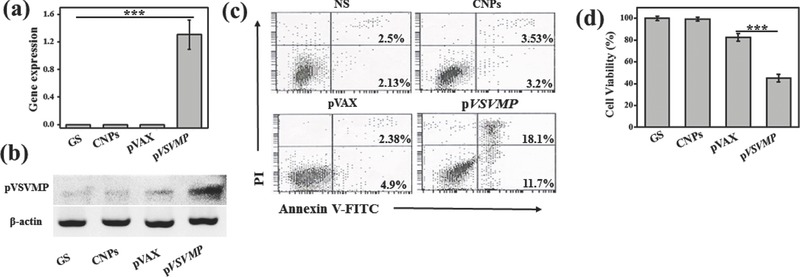
Anti‐tumor efficiency of DNA nanocomplex in vitro. Gene expression of VSVMP in SKOV3 ovarian cancer cells after being treated by p*VSVMP* nanocomplex was confirmed by both a) RT‐PCR and b) western blot. c) SKOV3 cells were treated with *VSVMP* gene to induce apoptosis. Early apoptosis cells (annexin V^+^ PI^−^) appear in the lower right quadrants. Late apoptosis cells (annexin V+ PI+) are in the upper right quadrants. d) After treatments with p*VSVMP* nanocomplex, pVAX nanocomplex, CNPs, or GS, the cell viability of SKOV3 ovarian cancer cells was measured by MTT assay. The p*VSVMP* nanocomplex significantly declined the cell viability. ***P < 0.001.

To evaluate the anticancer activity of p*VSVMP* nanocomplex in vitro, real‐time reverse transcriptase polymerase chain reaction (RT‐PCR) and western blot analysis were performed to confirm the gene expression of VSVMP in SKOV3 cells. The cells in p*VSVMP* nanocomplex treated group expressed a high level of mRNA of VSVMP while the expression of other groups was negligible (**Figure** **3**a). Western blot showed a similar result that p*VSVMP* nanocomplex group had an obvious VSVMP protein band compared to other groups (Figure 3b), indicating that p*VSVMP* nanocomplex group can efficiently express VSVMP in ovarian cancer cells. The tumor cell apoptosis induced by p*VSVMP* nanocomplex was investigated by annexin V‐FITC and PI double staining (Figure 3c). Annexin V^+^ PI^−^ cells (lower right quadrants) represent early apoptosis cells and annexin V^+^ PI^+^ cells (upper right quadrants) represent late apoptosis cells. The percentage of cells in each of these two quadrants was recorded. The flow cytometry results showed that p*VSVMP* nanocomplex caused 29.8% of apoptotic cells, but the apoptotic proportions caused by pVAX nanocomplex (7.28%, controlled pDNA) and CNPs (6.73%) were much lower. As shown in Figure 3d, p*VSVMP* nanocomplex caused 55.0% ± 3.4% growth inhibition of SKOV3 cell while growth inhibition caused by pVAX nanocomplex and CNPs was 17.5% ± 3.6% and 1.0% ± 1.7%, respectively. All these results demonstrated that the p*VSVMP* nanocomplex could efficiently express VSVMP in SKOV3 cells, resulting in the apoptosis of cancer cells.

### 2.3 Biodistribution and Acute Toxicity Test on Mice

**Figure 4:**
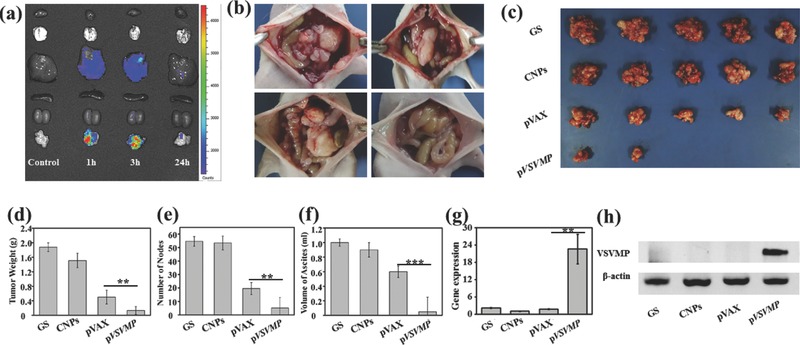
Anti‐tumor effect of DNA nanocomplex in vivo. a) Biodistribution of CNPs labeled by coumarin‐6. Coumarin‐6‐drived fluorescence in tumor was much higher than that in other critical organs. The total fluorescence was weakening over time and the majority was gone 24 h later. b) The representative photographs of intraperitoneal metastatic nodes from GS group (upper left), CNPs group (upper right), pVAX group (lower left), and p*VSVMP* group (lower right). c) The harvested tumor nodes in each group; d) tumor weight, e) number of nodes, and f) ascites volume of the mice in each group were recorded, and remarkable antitumor efficiency caused by the p*VSVMP* nanocomplex was observed. VSVMP expression of tumor nodes in p*VSVMP* group was confirmed by g) RT‐PCR and h) western blot.**P < 0.01 and ***P < 0.001.

To evaluate the biodistribution of CNPs, we labeled CNPs by coumarin‐6 as a fluorescent probe. The critical organs and tumor nodes of mice were harvested (**Figure** **4**a). Coumarin‐6‐drived fluorescence was much higher in tumor nodes than other critical organs. The fluorescence in tumor sites was diminishing over time and nearly vanished after 24 h. Meanwhile the acute toxicity test was conducted on mice. Through intravenous injection, the maximum tolerated dose of CNPs was 900 mg kg^−1^. While the tolerance dose of CNPs was 1 × 10^4^ mg kg^−1^, as 1.5 thousand folds as the therapeutic dose was higher through intraperitoneal injection than intravenous injection. These results implied that the DNA nanocomplex is very safe especially through intraperitoneal injection, and can markedly enhance the tumor accumulation.

### 2.4 In Vivo Anticancer Effect of the DNA Nanocomplex

To assess the anticancer activity of p*VSVMP* nanocomplex on the intraperitoneal metastatic tumor model of SKOV3 ovarian carcinoma, the tumor‐bearing mice were divided into four groups and were treated with p*VSVMP* nanocomplex, pVAX nanocomplex, CNPs, and glucose solution (GS), respectively. Remarkable effect was observed from the representative images and the mice treated with p*VSVMP* nanocomplex suffered from smaller tumor burden than other groups (Figure 4b,c). After that, tumor nodules in each group were harvested and weighed. The average tumor weight of mice treated with p*VSVMP* nanocomplex was 0.13 ± 0.11g, while that of those treated with pVAX nanocomplex, CNPs, and GS were 0.50 ± 0.19g, 1.51 ± 0.20 g, and 1.88 ± 0.12 g, respectively (Figure 4d). Figure 4e shows the number of tumor nodes of each group. Obviously, p*VSVMP* nanocomplex induced a dramatic growth suppression of tumor in mice (*P* < 0.01, vs pVAX group). Meanwhile it showed a significant reduction in the ascites volume of the p*VSVMP* nanocomplex‐treated mice, compared with that of other groups (Figure 4f). The volume of ascites of mice was 0.05 ± 0.2 mL in p*VSVMP* nanocomplex group, 1.00 ± 0.05 mL in GS group, 0.90 ± 0.1 mL in CNPs group, and 0.60 ± 0.08 mL in pVAX group. Likewise, a large amount of hemorrhagic ascites could be observed in the pVAX, CNPs, and GS groups, while the p*VSVMP* nanocomplex treated group was in normal state. These results demonstrated that intraperitoneal injection of p*VSVMP* nanocomplex could efficiently inhibit the tumor growth and ascites formation of SKOV3 ovarian carcinoma. RT‐PCR (Figure 4g) and western blot (Figure 4h) analysis were conducted on the tumor tissues. Similarly, the expression level of VSVMP was significantly high in vivo. All this demonstrated that the p*VSVMP* nanocomplex can efficiently inhibit the growth of ovarian cancer through expressing the VSVMP protein in a high level.

**Figure 5:**
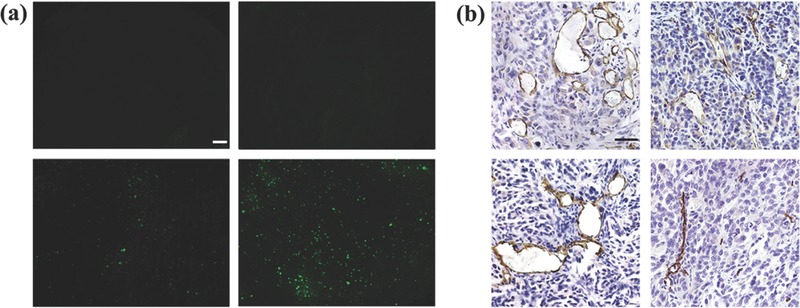
Anti‐tumor mechanism analysis. a) TUNEL staining analysis of tumor nodes. A large number of cells with green fluorescence were observed in p*VSVMP* group (lower right), identified as apoptotic tumor cells, whereas such a phenomenon was rare in GS group (upper left), CNP group (upper right), and pVAX group (lower left). Scale bar, 50 µm. b) CD31 staining was performed to assess the anti‐angiogenesis effect of VSVMP protein on ovarian carcinoma in GS group (upper left), CNP group (upper right), pVAX group (lower left), and p*VSVMP* group (lower right). Scale bar, 50 µm.

To investigate the mechanism of anticancer activity of p*VSVMP* nanocomplex in vivo, the terminal deoxynucleotidyl transferase‐mediated dUTP nick end‐labeling (TUNEL) assays were carried out. A large number of dots with green fluorescence (identified as apoptotic cell) could be observed in the tumor tissues treated with p*VSVMP* nanocomplex, while it rarely happened in other groups (**Figure** **5**a). These implied that induction of apoptosis might be involved in the mechanisms of inhibition of ovarian cancer by p*VSVMP* nanocomplex.

Moreover, tumor tissues from each treatment group were stained with anti‐CD31 antibody to evaluate whether the p*VSVMP* nanocomplex could inhibit tumor angiogenesis. Figure 5b shows that the microvessel density characterized by CD31 positive staining was significantly attenuated in p*VSVMP* nanocomplex group compared with other groups. The data suggested that anti‐angiogenesis may be another mechanism for suppressing ovarian cancer by p*VSVMP* nanocomplex in vivo.

**Figure 6:**
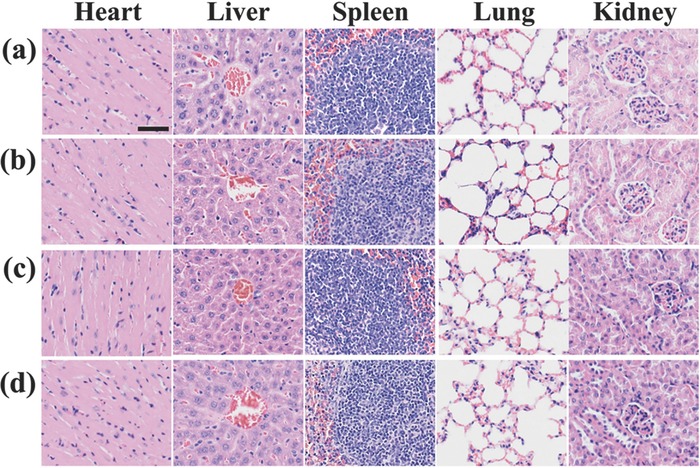
Systemic toxicity analysis. After being treated with a) GS, b) CNPs, c) pVAX nanocomplex, and d) p*VSVMP* nanocomplex, critical organs (heart, liver, spleen, lungs, and kidneys) were harvested and H&E staining was used to assess the systemic toxicity. Scale bar, 50 µm.

During the period of animal experiment, we did not find any decrease in physical activity and any increase in eye discharge of mice in p*VSVMP* nanocomplex group. Before the sacrifice, nearly all the mice suffered cachexia except for the mice in p*VSVMP* nanocomplex group. Hematoxylin and eosin (H&E) staining showed that the DNA nanocomplex did not cause obvious systemic toxicity (**Figure** **6**).

## 3 Discussion

Gene therapy has great promise for cancer treatment.[[qv: 6d]] In this work, we designed a VSV‐inspired p*VSVMP* nanocomplex for ovarian cancer therapy. Our results indicated that this p*VSVMP* nanocomplex could efficiently express *VSVMP* into ovarian cancer cells and induce cell death in vitro. Moreover, intraperitoneal administration of p*VSVMP* nanocomplex effectively inhibited the intraperitoneal metastasis of ovarian cancers in vivo, without causing significant systemic toxicity. These results demonstrated that the VSV‐inspired p*VSVMP* nanocomplex has a potential clinical application in ovarian cancer gene therapy.

Oncolytic viruses have promising application in cancer therapy,^13^ while the clinical applications are restricted by virus‐associated safety issues.[[qv: 5a,14]] Previously, many attempts have been performed to reduce the virus‐associated safety issues by virus modification.^15^ In this work, we attempted to resolve the virus‐associated safety issues. Inspired by the fact that VSVMP plays a critical role in VSV‐induced cancer cell death, we constructed a p*VSVMP* nanocomplex to mimic VSV to kill cancer cells. Results indicated that p*VSVMP* nanocomplex can efficiently express VSVMP in cancer cells, leading to the cancer cell death in vitro and in vivo. Therefore, it is an interesting method to use a DNA nanocomplex to express a critical component of oncolytic virus for cancer therapy, which could also inspire the development of future oncolytic virus‐inspired cancer gene therapies.

Multi‐target anticancer drugs attract more and more attention recently. In the past, many gene therapies were designed to target a single therapeutic target. However, single‐target drug treated cancers tend to recur frequently and develop resistance.^16^ Currently, studies show that drugs targeting multiple pathways of cancer simultaneously can generate synergetic effect and achieve durable therapeutic effects.^17^ In this work, we designed a p*VSVMP* nanocomplex to express VSVMP for cancer therapy. The VSVMP can inhibit the host gene transcription,^9^ the nucleocytoplasmic transport of host RNAs and proteins,^18^ the Akt/protein kinase B signaling,^19^ and the vascular endothelial growth factor secretion.^20^ Therefore, this p*VSVMP* nanocomplex that targets multi‐targets can avoid some potential drawbacks of conventional single‐target gene therapy.

Currently, the lack of efficient and safe gene delivery system is a great challenge for gene therapy.[[qv: 10a,21]] In this work, the designed VSV‐inspired DNA nanocomplex consists of cationized MPEG‐PLA(CNPs) nanoparticles and the VSVMP plasmid. The CNPs are assembled by MPEG‐PLA and DOTAP. Previously, MPEG‐PLA micelle‐enabled novel drugs have been approved for clinical use,^22^ and some DOTAP based cationic liposomal products have been approved for clinical trials as well.[[qv: 10a,23]] The use of a well‐studied MPEG‐PLA and DOTAP as the component can benefit to promise the nanoparticles with future clinical applications. The content of cationic DOTAP in the CNPs is much lower than that in past cationic liposomes. Meanwhile, the PEGylated surface could also contribute to reducing the cytotoxicity of CNPs.^24^ So, these CNPs have a low cytotoxicity (IC_50_ > 300 µg mL^−1^). Importantly, it can efficiently transfect DNA into ovarian cancer cells, and the transfection ability is as high as that of conventional virus vector.^25^ Moreover, the CNPs can be readily prepared, which promise the future large‐scale production. Therefore, the CNPs are a kind of low‐cytotoxic and high‐efficient gene delivery system for ovarian cancer gene therapy.

Intraperitoneal metastasis is a common event of ovarian cancer,^26^ and the intraperitoneal administration of drugs is often used to treat the intraperitoneal metastasis of ovarian cancer in clinic.^27^ Intraperitoneal administration of drugs shows great pharmacokinetic advantage for tumors could be directly exposed to high concentration of drugs.^28^ Our data showed that the intraperitoneal administrated DNA nanocomplex could efficiently distribute into the tumor nodes in abdominal cavity (Figure 4a). Meanwhile, intraperitoneal administration of p*VSVMP* nanocomplex effectively inhibits the intraperitoneal metastasis of ovarian cancers in vivo, and the tumor inhibition rate is as high as 93%. Furthermore, we confirmed that the tolerance dose of intraperitoneal administrated CNPs in mice is as high as 10^4^ mg kg^−1^. Under therapeutic dose, the p*VSVMP* nanocomplex does not cause obvious pathological changes in major organs. Therefore, p*VSVMP* nanocomplex has potential clinical application in ovarian cancer therapy.

## 4 Experimental Section

*Materials and Plasmids*: Monomethoxy poly (ethylene glycol)‐poly (d,l‐lactide) (MPEG_2000_‐PLA_2000_, MW 4000), a diblock copolymer, was synthesized in the lab.^29^ Branched polyethylenimine (PEI, average molecular weight 25 kDa), *N*‐[1‐(2,3‐dioleoyloxy) propyl]‐*N,N,N*‐trimethylammonium chloride (DOTAP), MTT, Dulbecco's modified Eagle's medium (DMEM), and RPMI‐1640 medium were purchased from Sigma‐Aldrich (USA). The therapeutic gene (*VSVMP*) was previously constructed into pVAX expression vector (p*VSVMP*) in the lab,^30^ and pVAX was used as the empty vector.

*Cell Culture*: SKOV3, HeLa, A549, and MB49 cell lines were obtained from the ATCC (American Tissue Culture Collection, USA). Cells were cultured in DMEM or RPMI‐1640 medium supplemented with 10% fetal bovine serum, and incubated in a humidified atmosphere containing 5% CO_2_ at 37 °C.

*Animal Procedure*: Female BALB/c‐nude mice and BALB/c mice (six weeks old) were obtained from Beijing HFK Bio‐Technology Co. Ltd. (Beijing, China) and fed under specific pathogen‐free conditions at the laboratory animal room. Before the experiment, the mice were given one‐week adaptive phase to accommodate themselves to the environment. All animal procedures were approved and controlled by the local ethics committee and carried out in accordance with the Institutional Animal Care and Use guidelines.

*Preparation of the CNPs*: To prepare the CNPs, 18 mg MPEG‐PLA diblock copolymer and 2 mg DOTAP were dissolved in methylene dichloride (KeLong Chemicals, Chengdu, China), then the transparent film was formed by rotary evaporation under the condition of 60 °C for 30 min. Next, the lipid film was rehydrated in double‐distilled water to form the micelles by self‐assembly. The resultant micelles were adjusted to the final concentration of 2 mg mL^−1^ and stored at 4 °C for future use.

*Characterization of the CNPs and DNA Nanocomplex*: The size distribution and zeta potential of the CNPs and DNA nanocomplex were detected by dynamic light scattering using a Zetasizer Nano ZS (Malvern Instruments, Worcestershire, UK). The equilibration time was 2 min and test temperature was 25 °C during measurements. The morphology analysis of the CNPs and DNA nanocomplex was performed via transmission electron microscope (TEM) (H‐6009IV, Hitachi, Japan). Furthermore, the morphology of the DNA nanocomplex was examined by an AFM (SPI4000, SII NanoTechnology Inc., Japan).

*DNA Binding Ability*: CNP/DNA complexes (DNA nanocomplex) with different mass ratios (0:1 to 20:1) were mixed with loading buffer and electrophoresed on 1% (w/v) agarose gel containing ethidium bromide (0.5 mg mL^−1^) for 30 min at 100 V. The resultant bands corresponding to different mass ratios were detected and photographed using ChemiDoc Imagers (Bio‐RAD ChemiDoc XRS, USA).

*Cytotoxicity Assay*: MTT colorimetric assay was used to monitor cell viability, which was based on the ability of living cells to turn MTT (a yellow, water‐soluble monotetrazolium salt) into water‐insoluble purple formazan that could be dissolved in dimethyl sulfoxide (DMSO). The concentration of CNPs or PEI25K that varied from 0 to 400 µg mL^−1^ was incubated with SKOV3 ovarian cancer cells at 37 ˚C for 48 h. Then, each well was supplemented with 20 µL MTT solution (5 mg mL^−1^) and incubated for another 4 h. Next, the medium was removed, and 150 µL DMSO was used to dissolve the purple formazan. Cell viability was expressed as percentage of absorbance in comparison with that of the control. The cytotoxicity of CNPs and PEI25K to 293T cells (embryonic kidney epithelial cells) was performed in the same way. Further the cytotoxicity and morphologic changes of SKOV3 cells and 293T cells treated by transfection concentration of CNPs (50 µg mL^−1^) or PEI25K (2 µg mL^−1^) were observed directly under microscope.

*Cellular Uptake of the DNA Nanocomplex*: To investigate the cellular uptake of the DNA nanocomplex by SKOV3 ovarian cancer cells, YOYO‐1 was used to label the *VSVMP* plasmid following the manufacturer's instruction. SKOV3 cells were seeded at a density of 1.5 × 10^5^ cells per well in 12‐well culture plates and incubated for 24 h. The medium was replaced with serum‐free medium and treated with the nanocomplex containing 1 µg YOYO‐1 labeled p*VSVMP*. The LysoTracker Red (Thermo Fisher Scientific, USA) was added for 30 min before fixing the cells. After the incubation at designed time intervals (0.5, 2, 4, and 6 h), the cells were washed and incubated with 4′,6‐diamidino‐2‐phenylindole, dihydrochloride (DAPI) (Sigma Chem. Co., St. Louis, MO, US) for 15 min, and then fixed with 4% paraformaldehyde for 15 min and washed with phosphate buffer saline (PBS) three times. Finally, the cells were analyzed with Olympus FluoView FV1000.

*Transfection Efficiency Analysis of DNA Nanocomplex*: SKOV3 cells were seeded into 6‐well plates at a density of 2 × 10^5^ cells per well in complete medium (DMEM containing 10% fetal bovine serum (FBS)). Then the medium was replaced with fresh nonserum medium. Green fluorescence protein plasmid (pGFP, 2 µg per well) as a report gene was mixed with the materials in serum‐free medium. The mass ratios of CNPs/pGFP were from 15:1 to 30:1 and PEI25K/pGFP was 1/1. After incubation for 6–8 h, the medium was replaced with complete medium and the cells were incubated for additional 48 h. The transfected cells were subsequently observed under a fluorescence microscope (Carl Zeiss Microimaging Inc., Thornwood, NJ). The expression of green fluorescence protein was quantitated by flow cytometry (BD FACSCalibur, BD Biosciences, San Jose, CA). The other cell lines, such as HeLa, A549, and MB49, were performed gene transfection quantitation analysis as control groups.

*In Vitro Anticancer Activity of DNA Nanocomplex*: To evaluate the anticancer activity of DNA nanocomplex in vitro, the SKOV3 cells were seeded into 96‐well plates at a density of 4000 cells per well. Twenty‐four hour later, the cells were divided into four groups (GS, CNPs, pVAX, and p*VSVMP*) and treated with p*VSVMP* nanocomplex, pVAX nanocomplex, null CNPs, and 5% GS separately. Forty‐eight hour later, the MTT assay was implemented to measure the cell viability.

Apoptosis detection was conducted for further investigation. The SKOV3 cells were seeded into 6‐well plates at a density of 2 × 10^5^ cells for 24 h. Then cells in serum‐free medium were treated with p*VSVMP* nanocomplex (50 µg CNPs/2 µg p*VSVMP*), pVAX nanocomplex (50 µg CNPs/2 µg pVAX), CNPs (50 µg CNPs), or GS. Six to eight hour later, the medium was replaced with 2 mL of DMEM complete medium and cells were incubated for another 48 h. The quantitative evaluation of apoptotic cells stained by annexin V‐FITC/PI (FITC annexin V apoptosis detection kit I, BD Pharmingen) was detected by flow cytometry.

*Quantitative RT‐PCR and Western Blot Assay*: Real‐time reverse transcriptase quantitative polymerase chain reaction (RT‐PCR) was performed to confirm the expression of VSVMP both in vitro and in vivo. Before RT‐PCR, total RNA was extracted from the treated cells or tumor tissues using RNA simple total RNA kit (Tiangen, China) according to the manufacture's protocol. Then the RT‐PCR comprised three steps: (i) the RT of RNA into cDNA, (ii) the amplification of the cDNA by PCR (forward primer, 5'‐CGC GGA TCC ATC ATG AGT TCC TTA AAG AAG‐3'; reverse primer 5'‐CGG AAT TCT CAT TTG AAG TGG CTG ATA GAA TCC‐3'), and (iii) the detection and quantification of amplification products in real time. Glyceraldehyde‐3‐phosphate dehydrogenase was used to normalize the mRNA expression of VSVMP. Experiments were performed in triplicates.

Furthermore, western blot assay was used to verify the expression of VSVMP in SKOV3 cells after being transfected with p*VSVMP* nanocomplex. Briefly, the cells were harvested after being treated with p*VSVMP* nanocomplex, pVAX nanocomplex CNPs, and GS. In addition, the tumor nodes of each group were harvested and grinded to powder under the protection of liquid nitrogen. Then the total protein was extracted by radioimmunoprecipitation assay (RIPA) buffer with cocktail protein inhibitors. The protein concentration was quantified by BCA protein assay kit (Bio‐Rad Laboratories, Hercules, CA, USA). The protein was separated out by gel electrophoresis and electroblotted onto polyvinylidene diﬂuoride membrane. The membranes were blocked with tris‐buffered saline w/Tween 20 (TBST) buffer containing 5% bovine serum albumin (BSA) for 1 h at room temperature and then the membranes were incubated with primary antibody (anti‐VSVMP, prepared in the lab) at 4 ˚C overnight. The membranes were subsequently washed with TBST for three times and incubated with the secondary antibody (goat anti‐rabbit polyclonal IgG antibody, Thermo Fisher Scientific, USA) for 1 h at room temperature, and followed by visualizing and detection. β‐Actin (Santa Cruz, CA, USA) was used as the internal standard.

*Biodistribution and Acute Toxicity Test*: Four groups (four mice per group) of female BALB/c nude mice were used to evaluate the distribution of the CNPs in vivo. Coumarin‐6 was used to label CNPs. The mice of control group were administrated no drugs and treatment groups were administrated 200 µL coumarin‐6/CNPs via intraperitoneal injection at designed time interval of 1, 3, and 24 h. To study the biodistribution of CNPs in vivo, the critical organs (heart, liver, spleen, lungs, and kidneys) and tumor nodules of the representative mice were harvested and examined by the coumarin‐6 associated green fluorescence under live image analysis instrument (IVIS Lumina, Caliper Life Sciences).

Acute toxicity test was performed by intravenous injection of 200 µL high concentration of p*VSVMP* nanocomplex (from 70 to 150 mg mL^−1^) on BALB/c mice. In the meantime, the maximum tolerance dose through peritoneal injection was investigated; the mice were given 1 mL of high‐concentration p*VSVMP* nanocomplex (200 mg mL^−1^) twice for 24 h and the survival of the mice was recorded.

*In Vivo Anticancer Efficiency of DNA Nanocomplex*: The intraperitoneal metastatic tumor model was established on female BALB/c‐nude mice by intraperitoneal injection of 1 × 10^7^ SKOV3 cell suspension. Then the mice were randomly divided into four groups (*N* = 5) on day 7 and given the following treatments: p*VSVMP* nanocomplex (CNPS 5 mg kg^−1^, p*VSVMP* 0.2 mg kg^−1^), pVAX nanocomplex (CNPS 5 mg kg^−1^, pVAX 0.2 mg kg^−1^), CNPs (CNPS 5 mg kg^−1^), GS. The treatment was performed for seven times at an interval of 1 d. On day 37, all mice were sacrificed by cervical vertebra dislocation. Tumors and vital organs were gathered and fixed in 4% neutral paraformaldehyde or frozen in liquid nitrogen for future analysis. Ascites volume, tumor weight, and the number of nodules were recorded.

*Histological Analysis*: Tissues were fixed in 4% neutral paraformaldehyde for at least 48 h and then were embedded in paraffin. For safety evaluation of the nanocomplex, consecutive paraffin wax‐embedded tissue sections of vital organs (4–5 µm) were dewaxed, rehydrated, and stained with H&E. A commercially available TUNEL kit (Promega, Madison, WI) was used to analyze the apoptotic effects in intraperitoneal metastatic tumor of SKOV3 cells. This analysis was performed following the manufacturer's protocol. Microvessels were stained with anti‐CD31 antibody (1:100 dilution of a rabbit polyclonal antibody (ab28364), Abcam, Inc.) for immunohistochemical detection. The sections of tumor nodules were conducted according to standard procedures. Antigen retrieval was performed for 10 min and incubated with anti‐CD31 antibody overnight, and then incubated with secondary antibody conjugated to horseradish peroxidase (HRP) (ZSGB‐BIO, Beijing, China). Finally, 3,3‐diaminobenzidine substrate (DAB Kit, Maixin Bio, Fujian, China) was used for visualization.

*Statistical Analysis*: All data were presented as mean ± standard deviation. Student's *t*‐test (two‐tailed) was applied to analyze the significance of the difference. Significant differences between groups were indicated by *P < 0.05, **P < 0.01, and ***P < 0.001, respectively.

## Conflict of Interest

The authors declare no conflict of interest.
